# Ribosomal and non-ribosomal PCR targets for the detection of low-density and mixed malaria infections

**DOI:** 10.1186/s12936-019-2781-3

**Published:** 2019-04-30

**Authors:** Lara Cotta Amaral, Daniela Rocha Robortella, Luiz Felipe Ferreira Guimarães, Jean Ezequiel Limongi, Cor Jesus Fernandes Fontes, Dhelio Batista Pereira, Cristiana Ferreira Alves de Brito, Flora Satiko Kano, Taís Nóbrega de Sousa, Luzia Helena Carvalho

**Affiliations:** 1 Instituto René Rachou, Fundação Oswaldo Cruz, FIOCRUZ-MINAS, Belo Horizonte, MG Brazil; 2 Universidade Federal de Minas Gerais, Belo Horizonte, MG Brazil; 3 Universidade Federal de Uberlândia, Uberlândia, MG Brazil; 4 Hospital Júlio Müller, Universidade Federal do Mato Grosso, Cuiabá, Mato Grosso Brazil; 5 Centro de Pesquisas em Medicina Tropical de Rondônia (CEPEM), Porto Velho, Rondônia Brazil

**Keywords:** Malaria, Molecular diagnosis, PCR, Submicroscopic, Mixed-malaria infections

## Abstract

**Background:**

The unexpected high proportion of submicroscopic malaria infections in areas with low transmission intensity challenges the control and elimination of malaria in the Americas. The current PCR-based assays present limitations as most protocols still rely on amplification of few-copies target gene. Here, the hypothesis was that amplification of different plasmodial targets—ribosomal (*18S rRNA*) and non-ribosomal multi-copy sequences (Pvr47 for *Plasmodium vivax* and Pfr364 for *Plasmodium falciparum*)—could increase the chances of detecting submicroscopic malaria infection.

**Methods:**

A non-ribosomal real-time PCR assay targeting Pvr47/Pfr364 (*NR*-*qPCR*) was established and compared with three additional PCR protocols, two of them based on *18S rRNA* gene amplification (*Nested*-*PCR* and *R*-*qPCR*) and one based on Pvr47/Pfr364 targets (*NR*-*cPCR*). The limit of detection of each PCR protocol, at single and artificial mixed *P. vivax*/*P. falciparum* infections, was determined by end-point titration curves. Field samples from clinical (n = 110) and subclinical (n = 324) malaria infections were used to evaluate the impact of using multiple molecular targets to detect malaria infections.

**Results:**

The results demonstrated that an association of ribosomal and non-ribosomal targets did not increase sensitivity to detect submicroscopic malaria infections. Despite of that, artificial mixed-malaria infections demonstrated that the NR-qPCR was the most sensitive protocol to detect low-levels of *P. vivax*/*P. falciparum* co-infections. Field studies confirmed that submicroscopic malaria represented a large proportion (up to 77%) of infections among asymptomatic Amazonian residents, with a high proportion of infections (~ 20%) identified only by the NR-qPCR.

**Conclusions:**

This study presents a new species-specific non-ribosomal PCR assay with potential to identify low-density *P. vivax* and *P. falciparum* infections. As the majority of subclinical infections was caused by *P. vivax*, the commonest form of malaria in the Amazon area, future studies should investigate the potential of Pvr47/Pfr364 to detect mixed-malaria infections in the field.

**Electronic supplementary material:**

The online version of this article (10.1186/s12936-019-2781-3) contains supplementary material, which is available to authorized users.

## Background

Malaria is a worldwide public health concern that is present in roughly 90 countries, mainly in tropical and subtropical regions [1]. While *Plasmodium vivax* is the most widely distributed parasite causing malaria, *Plasmodium falciparum* accounts for the most severe forms of the disease [2]. Although malaria incidence rate is estimated to have decreased by 18% globally between 2010 and 2016, a recent increase in case incidence occurred in the Americas, particularly in the Amazon rainforest [1, 3].

In order to progress towards malaria control and elimination, it is critical to understand the sources of transmission (the infectious reservoir) and those at risk of infection at the population level [4]. In this context, the molecular detection of *Plasmodium* infections in endemic areas have confirmed previous finding of high frequencies of malaria infections at densities below the limit of conventional microscopic diagnostics [5–9]. It is particularly relevant as data from systematic reviews have shown that across different geographic areas low-density infections may represent the majority of malaria infections [10, 11]. Accordingly, a substantial proportion of asymptomatic and submicroscopic malarial infections has been described in peri-urban areas of the Brazilian Amazon [12]. Many are the implications of these findings as submicroscopic malaria carriers may be able to transmit the *Plasmodium* parasites, acting as reservoirs for malaria [13, 14]. Beyond the practical value of using molecular tools to identify submicroscopic carriers and mixed-species infections, malaria infections at any density may have significant health and socioeconomic consequences [15].

Historically, the small subunit 18S of the ribosomal RNA gene (*18S rRNA*) has been the most common target used for molecular diagnosis of malaria [16–20]. As this gene is present in few copies (5 to 8) in the genome of *Plasmodium* parasites [21], low sensitivity and reproducibility of standard PCR protocols based on *18S rRNA* gene amplification have been described [22, 23]. In the last decade, the genomic data mining of *Plasmodium* parasites has allowed the discovery of new species-specific multi-copy targets which show potential for molecular diagnosis of *P. vivax* and *P. falciparum* malaria [24–26]. Among the promising targets include the non-coding subtelomeric repeat sequences Pvr47 and Pfr364 that are present in 14 and 41 copies in the genomes of *P. vivax* and *P. falciparum*, respectively [24]. While there is evidence for their location and distribution, the biological functions of Pvr47 and Pfr364 remains to be established. By using a single-step PCR assay to amplify Pvr47/Pfr364 targets, it was possible to demonstrate the relatively higher sensitivity of these targets as compared to the amplification of *18S rRNA* gene by the conventional nested-PCR assay [24].

Since most malaria PCR-based protocols still relies on amplification of *18S rRNA* gene, which has low sensitivity to detect low-density infections, we evaluated here how useful Pvr47/Pfr364 targets are to detect single and mixed *P. vivax* and *P. falciparum* infections in clinical and subclinical malaria. As the original PCR protocol to amplify Pvr47/Pfr364 involved DNA visualization on gel electrophoresis [24], here a new qPCR protocol targeting these high-copy non-ribosomal sequences was established. The experimental approach evaluated whether amplification of different plasmodial targets (Pvr47/Pfr364 and *18S rRNA* gene) could increase the chances of detecting submicroscopic malaria infections. For that, field samples (clinical and subclinical malaria) were amplified by four different PCR assays, two of them targeting Pvr47/Pfr364 sequences [24] and two targeting the *18S rRNA* gene [16, 17].

## Methods

### Study population and participants

Ethical and methodological aspects of this study were approved by the Ethical Committee of Research on Human Beings from the René Rachou Institute/Oswaldo Cruz Foundation (protocols No 24/2008, and No 1.821.955/2016), according to the Brazilian National Council of Health (Resolutions 196/96 and 466/12). All participants were informed about the objectives and procedures of the study, with voluntary participation through written informed consent.

#### Clinical malaria

This group included individuals with clinical suspicion of malaria who sought care at Brazilian malaria reference healthcare facilities located in both endemic (States of Rondônia and Mato Grosso) and non-endemic areas (Minas Gerais). After short-trips to malaria transmission areas, these individuals presented symptoms suggestive of uncomplicated malaria infection, such as fever, myalgia, chills, and headaches. Non-inclusion criteria included: (i) refusal or inability to sign the informed consent; (ii) age below 5 years-old; (iii) pregnant women; and (iv) any other co-morbidity that could be traced. One-hundred-and-ten patients were enrolled in the study, which gives 90% statistical power at 5% significance level assuming 30% of prevalence by light microscopy and an estimative of 50% increase by molecular assays; the majority of study population were adults, with a median age of 40 years (IQR 30.75–48.25), and a proportion female:male of 1:5. For each individual, blood sample was collected at a single time point by venipuncture or finger prick. The period of blood collection varied from 2008 to 2017, and since there, all samples from participants have been maintained in the biorepository of malaria research group at FIOCRUZ-MINAS (Belo Horizonte, MG), Brazilian Ministry of Health, according to the local legislation.

#### Subclinical malaria

This group included malaria-exposed individuals who had participated of cross-sectional surveys carried-out between 2008 and 2015 in a rural community of the Brazilian Amazon rainforest, Rio Pardo (1°46′S—1°54′S, 60°22′W—60°10′W), Presidente Figueiredo municipality, State of Amazonas. The study site and malaria transmission patterns have been described in details elsewhere [27–29]. In this area, malaria transmission is considered hypo to mesoendemic, and the majority of residents were natives from the Amazon region [27]. For the current study, the non-inclusion criteria were: (i) refusal or inability to sign the informed consent; (ii) any signs and/or symptoms that could be related to symptomatic malaria such as fever, myalgia, chills, and headaches; the absence of symptoms was self-reported and obtained during a personal interview conducted through structured questionnaire, as previously described [27]; (iii) age below 5 years-old, as subclinical malaria infection is not prevalent in Amazon children [30]; (iv) pregnant women; and (v) any other morbidity that could be traced. This group was composed by 324 participants; considering 7% of prevalence by light microscopy [27] and assuming that in an area of greater chance of *P. vivax* infection molecular assays is expected to increase malaria prevalence by a factor of 2 [11], sample size will give more than 90% of statistical power at 5% significance level. The median age of studied individuals were 40 years (IQR 24–56), with female:male ratio of 1:1.5, and an average of 35 years (IQR 21–52) living in the endemic area. For each individual, blood sample was collected at a single time-point by venipuncture or finger prick. As in the study area the number of malaria cases fluctuated during the last 8 years, reflecting periods of high and low malaria transmission [31], samples covered the temporal-variation in the profile of malaria transmission; thus, the blood samples were collected from November 2008 to June 2015, and all biological specimens have been maintained in the biorepository of malaria research group at FIOCRUZ-MINAS (Belo Horizonte, MG), Brazilian Ministry of Health, according to the local legislation.

### Conventional light microscopy

At the time of blood collection, all individuals (clinical and subclinical groups) were submitted to a finger-prick for malaria diagnosis by light microscopy. The Giemsa-stained thick blood smears were prepared and examined by experienced local microscopists, according to the malaria diagnosis guidelines of the Brazilian Ministry of Health [32]. Parasite density was estimated as the number of parasites per microlitre of blood (parasites/µL), and all microscopically positive cases were treated immediately in local health services, following the national malaria treatment protocols recommended by the Brazilian Ministry of Health [33].

### Extraction of genomic DNA

**Fig. 1:**
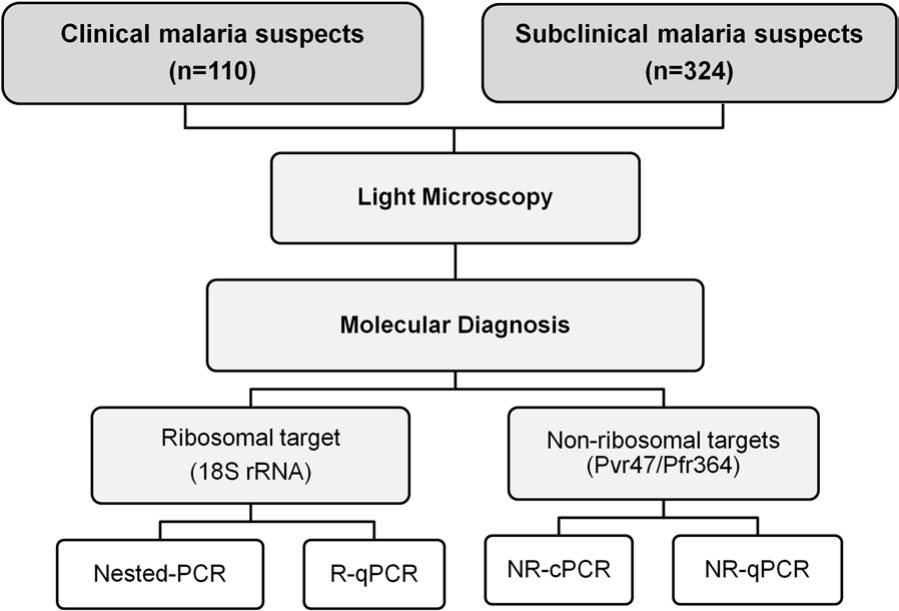
Methodological strategy for field evaluation of PCR-based protocols. Regardless the results obtained by light microscopy, blood-derived DNA samples from clinical (n = 110) and subclinical (n = 324) malaria suspects were submitted to species-specific PCR based-protocols targeting ribosomal (*18S rRNA* gene) and non-ribosomal *Plasmodium* sequences. The *18S rRNA*-based protocols included a Nested-PCR assay adapted from the original protocol (16) with modifications (22), and a real-time PCR assay (*R*-*qPCR*) as previously described (17). The non-ribosomal (NR) amplification of *P. vivax* (Pvr47) and *P. falciparum* (Pfr364) involved a previously described single step conventional PCR assay (*NR*-*cPCR*) (24), and a real-time PCR protocol (*NR*-*qPCR*) whose primers and cycling conditions were described in “Methods”

The experimental approach to the molecular detection of clinical and subclinical malaria infections was included in Fig. 1. Genomic DNA (gDNA) was extracted from either whole blood samples collected in EDTA, or from dried blood spots on filter paper using the Gentra Puregene Blood Kit (Qiagen) and the QIAamp DNA Mini Kit (Qiagen), respectively, according to manufacturer’s instructions. As an internal control of the DNA extractions, 10% of the samples extracted were randomly submitted to a PCR assay for the amplification of a human gene (ABO blood group), according to the protocol previously described [34]. All samples tested amplified the target gene. The extracted DNA was stored at − 20 °C until use.

### Panel of *Plasmodium* reference samples

The following parasites were used as reference in molecular assays: (i) *P. falciparum* (3D7 strain) from in vitro continuous blood-stage cultures maintained in the routine of the laboratory, according to protocol previously described [35]; (ii) *P. vivax, P. falciparum* and *Plasmodium malariae* from peripheral blood of malaria infected individuals whose species-specific diagnosis was confirmed by PCR protocols (Malaria Biorepository, FIOCRUZ-Minas, MG, Brazil); (iii) *Plasmodium brasilianum*/*P. malariae* (Peruvian III strain, MR4-349) kindly provided by the Malaria Research and Reference Reagent Resource Center, MR4 (Biodefense and Emerging Infections Research Resources Repository, BEI Resources, NIAID/NIH, ATCC, USA).

### End-point *Plasmodium* DNA titrations of single and mixed *Plasmodium vivax* and *Plasmodium falciparum* infections

DNA samples from high-density *P. vivax* (12,900 parasites/µL) and *P. falciparum* (13,400 parasites/µL) infections were used to determine the end-point detection for ribosomal and non-ribosomal PCR assays. For each plasmodial DNA, three-fold dilutions were prepared range from 2000 to 0.3 parasites/µL. Similarly, artificial mixed malaria infections were produced by mixed DNA samples from *P. vivax* and *P. falciparum* in different proportions (1:1 until 1:2000; threefold dilutions). Each sample was assayed in triplicate and submitted to all PCR protocols (Fig. 1).

### Primers design and real-time PCR conditions to amplify the non-ribosomal Pvr47/Pfr364 targets (NR-qPCR)

Primers and MGB (minor groove binder) probes were designed for Pvr47 and Pfr364 targets using Primer-Blast (NCBI) and OLIGO (version 4.0, 1999) softwares, considering conserved regions in each species alignments. The alignments were based on the sequences previously described [24], composed of 14 copies of Pvr47 (*P. vivax* Sal-I strain) and 41 copies of Pfr364 (subfamilies 1 and 2 of *P. falciparum* 3D7 strain). The set of oligonucleotides obtained for Pvr47 was 5′TCCGCAGCTCACAAATGTTC3′ (*forward*), 5′ACATGGGGATTCTAAGCCAATTTA3′ (*reverse*), and 5′HEX-TCCGCGAGGGCTGCAA3′ (probe), which binds respectively to positions 142–161, 245–222 and 189–204 of *P. vivax* Sal-I (GenBank accession number AAKM01000578), resulting in a 104 bp amplicon. For Pfr364, the set used was 5′ACTCGCAATAACGCTGCAT3′ (*forward*), 5′TTCCCTGCCCAAAAACGG3′ (*reverse*), and 5′FAM-GGTGCCGGGGGTTTCTACGC3′ (probe), which binds respectively to positions 313–331, 400–383 and 335–354 of *P. falciparum* 3D7 (*Pf3D7_04_12625_14152*, in [24]), resulting in an 88 bp amplicon. All NR-qPCR reactions were performed in 10 μL volumes containing 2 μL of DNA (~ 3 to 6 μL of whole blood) and 5 μL of TaqMan Universal PCR Master Mix (Thermo Fisher Scientific). For Pvr47 amplification was used 50 nM of forward primer, 900 nM of reverse primer, and 250 nM of probe; for Pfr364, 900 nM of forward primer, 300 nM of reverse primer, and 150 nM of probe were used. The PCR assays were performed using the automatic thermocycler ViiA7 Real-Time PCR System (Thermo Fisher Scientific) and the following cycling parameter: a pre-incubation and initial denaturation, respectively, at 50 °C for 2 min and 95 °C for 10 min, followed by 40 cycles of denaturation at 95 °C for 15 s, primers annealing at 52 °C for 1 min, and extension at 60 °C for 1 min. The fluorescence acquisition was performed at the end of each extension step. Analytical sensitivity and specificity of Pvr47 and Pfr364 NR-qPCR assays were included as an Additional file 1. Specificity was investigated using template DNA from either malaria unexposed individuals (n = 30) or other *Plasmodium* species (Additional file 2). For the limit of detection, standard curves were prepared from serial dilution of plasmid DNA carrying the Pvr47 or Pfr364 target (Additional file 3). The cycle threshold (Ct) values of 37 and 38 (*C*_*t*_≤ 37 or *C*_*t*_≤ 38) were used to define positivity to *P. vivax* and *P. falciparum*, respectively.

### Amplification of Pvr47/Pfr364 targets by conventional PCR assay (NR-cPCR)

The amplification of the targets Pvr47 (*P. vivax*) and Pfr364 (*P. falciparum*) were conducted by using primers previously described [24]. Details about primers and cycling conditions were included in Additional file 4.

## *18S rRNA* gene amplification by nested-PCR (Nested-PCR) and real-time PCR assay (R-qPCR)

Nested-PCR assay for amplification of *18S rRNA* gene was performed as described [22], using genus and species-specific primers previously described [16]. The real-time PCR (R-qPCR) method uses a single pair of genus-specific primers for the detection of the *18S rRNA* gene, with two internal species-specific hydrolysis probes for *P. falciparum* and *P. vivax* [17]. Primers and cycling conditions for *18SrRNA* gene amplification (Nested-PCR and R-qPCR) were included as Additional file 4.

### Statistical analysis

Differences in proportions were evaluated using the Chi square (χ^2^) test or Fisher’s exact test, as appropriate. The Probit Regression analysis was used to define the limit of detection (LOD) of NR-qPCR assay, with different input concentrations of plasmid DNA used to calculate the predicted proportion of positive results (MedCalc Statistical Software program, Ostend, Belgium). Heat map of malaria prevalence was constructed using the heatmap.2 function available in the R package gplots. The assessment of sensitivity and specificity of PCR assays was determined as described [23], with the reference standard established by combining the detection of any PCR assay excluding the protocol under evaluation. The analyses of sensitivity and specificity were performed in the *GraphPad InStat*, version 3.0 (GraphPad Software, San Diego, CA, USA). The significance at the 5% level was considered to all analysis.

## Results

### Limit of detection of Pvr47 and Pfr364 NR-qPCR

To determine the LOD of NR-qPCR protocols, standard curves were constructed for each DNA plasmid carrying the target gene (Pvr47 or Pfr364) through serial dilution ranging from 20,000 to 0.05 copies/μL (Additional file 3). By using probit regression analysis was possible to demonstrate that NR-qPCR developed here presented a 95% probability of detecting levels as low as 0.66 copies/µL for *P. vivax* (Additional file 5A) and 3.27 copies/µL for *P. falciparum* (Additional file 5B). No amplification was observed by using template DNA from either malaria unexposed individuals or other *Plasmodium* species (data not shown, Additional file 2).

### Ribosomal and non-ribosomal detection of monoinfections caused by either *P. vivax* or *P. falciparum*

The ability of different targets (*18S rRNA* vs. Pvr47/Pfr364) to detect *P. vivax* and *P. falciparum* monoinfections at low parasite densities was investigated through serial dilutions of field samples containing known amounts of either *P. vivax* or *P. falciparum* gDNA. In case of *P. vivax* monoinfection, no significant difference was observed between parasite targets amplified by PCR (Table 1). Despite of that, the NR-qPCR was the only protocol able to consistently detect the lowest levels of parasite densities (all replicates amplified until 1 parasite/µL). Variability between PCRs assays using the same target resulted in a difference in positivity between conventional and real-time PCR assays targeting Pvr47 (67% vs. 96% for NR-cPCR and NR-qPCR, respectively). Considering *P. falciparum* titration (Table 2), regardless the PCR assay, the amplification of Pfr364 sequence was more precise than *18S rRNA* gene (80% vs. 59%, *p *= 0.0359, Fisher’s exact test). While the amplification of Pfr364 by NR-qPCR was reproducible until 3 parasites/μL, the amplification of *18S rRNA* was inconsistent, alternating between positive and negative results at similar levels of parasite density (both nested-PCR and R-qPCR).

**Table 1:** Titration of *P. vivax* single infection by PCR assays targeting ribosomal (*18S rRNA*) and non-ribosomal (Pvr47) species-specific sequences

*P. vivax* monoinfection (12,900 parasites/µL)^c^				
Parasite density (µL)^d^	18S rRNA		Pvr47	
	Nested-PCR	R-qPCR	NR-cPCR	NR-qPCR
2000	3/3 (100%)	3/3 (100%)	3/3 (100%)	3/3 (100%)
670	3/3 (100%)	3/3 (100%)	3/3 (100%)	3/3 (100%)
220	3/3 (100%)	3/3 (100%)	3/3 (100%)	3/3 (100%)
74	3/3 (100%)	3/3 (100%)	3/3 (100%)	3/3 (100%)
25	3/3 (100%)	3/3 (100%)	3/3 (100%)	3/3 (100%)
8	3/3 (100%)	3/3 (100%)	3/3 (100%)	3/3 (100%)
3	3/3 (100%)	3/3 (100%)	0/3 (0%)	3/3 (100%)
1	1/3 (33%)	2/3 (67%)	0/3 (0%)	3/3 (100%)
0.3	0/3 (0%)	2/3 (67%)	0/3 (0%)	2/3 (67%)
PCR positivity	22/27 (81%)^a,b^	25/27 (93%)^b^	18/27 (67%)^a^	26/27 (96%)^b^
Target positivity	47/54 (87%)^a′^		44/54 (81%)^a′^	

*P. vivax* blood-derived DNA template was serial diluted (2000 to 0.3 parasites/µL) and submitted to each PCR protocol in triplicate. The results were expressed as the number of positive samples in relation to the total of replicates (percentage of positive). PCR assays were defined as described in legend of Fig. 1

Different letters (a,b) indicate differences between proportions (*p *< 0.05, Fisher’s Exact Test)

No difference was observed between proportions of targets positivity (a′)

^c^Determined by Light Microscopy

^d^Parasite density (µL of blood) was estimated according to the fold-dilution

**Table 2:** Titration of *P. falciparum* single infection by PCR assays targeting ribosomal (*18S rRNA*) and non-ribosomal (Pfr364) species-specific sequences

*P. falciparum* monoinfection (13,400 parasites/µL)^c^				
Parasite density (µL)^d^	18S rRNA		Pfr364	
	Nested-PCR	R-qPCR	NR-cPCR	NR-qPCR
2000	3/3 (100%)	3/3 (100%)	3/3 (100%)	3/3 (100%)
670	3/3 (100%)	3/3 (100%)	3/3 (100%)	3/3 (100%)
220	3/3 (100%)	3/3 (100%)	3/3 (100%)	3/3 (100%)
74	3/3 (100%)	3/3 (100%)	3/3 (100%)	3/3 (100%)
25	2/3 (67%)	2/3 (67%)	3/3 (100%)	3/3 (100%)
8	1/3 (33%)	1/3 (33%)	3/3 (100%)	3/3 (100%)
3	1/3 (33%)	1/3 (33%)	2/3 (67%)	3/3 (100%)
1	0/3 (0%)	0/3 (0%)	1/3 (33%)	0/3 (0%)
0.3	0/3 (0%)	0/3 (0%)	0/3 (0%)	1/3 (33%)
PCR positivity	16/27 (59%)^a^	16/27 (59%)^a^	21/27 (78%)^a^	22/27 (81%)^a^
Target positivity	32/54 (59%)^a′^		43/54 (80%)^b′^	

*P. falciparum* blood-derived DNA template was serial diluted (2000 to 0.3 parasites/µL) and submitted to each PCR protocol in triplicate. The results were expressed as the number of positive samples in relation to the total of replicates (percentage of positive)

Different letters (a, a′, b′) indicate differences between proportions (*p *< 0.05, Fisher’s Exact Test). PCR assays were defined as described in legend of Fig. 1

^c^Determined by light microscopy

^d^Parasite density (µL of blood) was estimated according to the fold-dilution

### Ribosomal and non-ribosomal detection of artificial mixed *P. vivax* and *P. falciparum* infections

Next, the ability of ribosomal and non-ribosomal targets to amplify artificial mixed malaria infections was evaluated. By fixing the amount of *P. vivax* DNA (1433 parasites/μL) and varying the amount of *P. falciparum* (1489 to 0.7 parasites/μL), it was possible to demonstrate that the protocols based on non-ribosomal targets were much more precise to identify both parasite species, even when *P. falciparum* was present at very low densities (Table 3). Taken together, non-ribosomal protocols identified 85% (41 out of 48) artificial mixed infections, while ribosomal protocols identified only 31% (15 out of 48). By comparing the variation intra-target, the amplification of *18S rRNA* gene by R-qPCR demonstrated a trend to amplify *P. vivax* in detriment of *P. falciparum*, even when *P. falciparum* DNA was present at relatively high concentrations (Table 3). By fixing the concentration of *P. falciparum* (1489 parasites/μL) and varying *P. vivax* densities (1433 to 0.7 parasites/μL), a good performance of both targets was observed until 6 parasites/μL (Table 4). Although there was no significant difference between the amplification of ribosomal and non-ribosomal targets (69% vs. 83%), the NR-qPCR protocol developed here seems to present a better performance as compared to *18S rRNA* amplification, detecting almost all replicates in all *P. vivax/P. falciparum* dilution points. In fact, NR-qPCR was the only PCR assay able to consistently detect mixed infection when one of the species was present in a ratio of about 700-fold lower than the other species (1489 vs. 2 parasites/μL to all replicates) (Table 4).

**Table 3:** Titration of *P. falciparum* in artificial mixed infections by PCR assays targeting ribosomal (*18S rRNA*) and non-ribosomal (Pvr47/Pfr364) species-specific sequences

Parasite density (per µl of blood)		18S rRNA						Pvr47/Pfr364 (NR targets)					
		Nested-PCR			R-qPCR			NR-cPCR			NR-qPCR		
Pv	Pf	#1	#2	#3	#1	#2	#3	#1	#2	#3	#1	#2	#3
1433	1489	Pv + Pf	Pv + Pf	Pv + Pf	Pv + Pf	Pv	Pv + Pf	Pv + Pf	Pv + Pf	Pv + Pf	Pv + Pf	Pv + Pf	Pv + Pf
	496	Pv + Pf	Pv + Pf	Pv + Pf	Pv	Pv	Pv	Pv + Pf	Pv + Pf	Pv + Pf	Pv + Pf	Pv + Pf	Pv + Pf
	165	Pv + Pf	Pv + Pf	Pv + Pf	Pv	Pv	Pv	Pv + Pf	Pv + Pf	Pv + Pf	Pv + Pf	Pv + Pf	Pv + Pf
	55	Pv	Pv + Pf	Pv + Pf	Pv	Pv	Pv	Pv + Pf	Pv + Pf	Pv + Pf	Pv + Pf	Pv + Pf	Pv + Pf
	18	Pv + Pf	Pv	Pv + Pf	Pv	Pv	Pv	Pv + Pf	Pv + Pf	Pv + Pf	Pv + Pf	Pv + Pf	Pv + Pf
	6	Pv	Pv	Pv	Pv	Pv	Pv	Pv + Pf	Pv + Pf	Pv + Pf	Pv + Pf	Pv + Pf	Pv + Pf
	2	Pv	Pv	Pv	Pv	Pv	Pv	Pv	Pv	Pv	Pv	Pv + Pf	Pv + Pf
	0.7	Pv	Pv	Pv	Pv	Pv	Pv	Pv + Pf	Pv	Pv	Pv + Pf	Pv + Pf	Pv
PCR positivity		13/24 (54%)^b^			2/24 (8%)^a^			19/24 (79%)^b,c^			22/24 (92%)^c^		
Target positivity		15/48 (31%)^a′^						41/48 (85%)^b′^					

Artificial mixtures containing *P. vivax* (*Pv*) and *P. falciparum* (*Pf*) in different proportions were prepared from well-characterized field samples, as described in Methods. The results are expressed as positive to *P. vivax* and *P. falciparum* (*Pv *+ *Pf*)*, P. vivax* (*Pv*) or *P. falciparum* (*Pf*). For each PCR assay, dilution points were assayed in triplicate (#1 to #3)

Different letters (a–c or a^′^, b^′^) indicate significant differences between proportions (*p *< 0.05, Fisher’s Exact Test)

**Table 4:** Titration of *P. vivax* in artificial mixed infections by PCR assays targeting ribosomal (*18S rRNA*) and non-ribosomal (Pvr47/Pfr364) species-specific sequences

Parasite density (per µl of blood)		18S rRNA						Pvr47/Pfr364 (NR targets)					
		Nested-PCR			R-qPCR			NR-cPCR			NR-qPCR		
Pf	Pv	#1	#2	#3	#1	#2	#3	#1	#2	#3	#1	#2	#3
1489	1433	Pv + Pf	Pv + Pf	Pv + Pf	Pv	Pv + Pf	Pv + Pf	Pv + Pf	Pv + Pf	Pv + Pf	Pv + Pf	Pv + Pf	Pv + Pf
	478	Pv + Pf	Pv + Pf	Pv + Pf	Pv + Pf	Pv + Pf	Pv + Pf	Pv + Pf	Pv + Pf	Pv + Pf	Pv + Pf	Pv + Pf	Pv + Pf
	159	Pv + Pf	Pv + Pf	Pv + Pf	Pv + Pf	Pv + Pf	Pv + Pf	Pv + Pf	Pv + Pf	Pv + Pf	Pv + Pf	Pv + Pf	Pv + Pf
	53	Pv + Pf	Pv + Pf	Pv + Pf	Pv + Pf	Pv + Pf	Pv + Pf	Pv + Pf	Pv + Pf	Pv + Pf	Pv + Pf	Pv + Pf	Pv + Pf
	18	Pf	Pv + Pf	Pv + Pf	Pv + Pf	Pv + Pf	Pv + Pf	Pv + Pf	Pv + Pf	Pv + Pf	Pv + Pf	Pv + Pf	Pv + Pf
	6	Pv + Pf	Pv + Pf	Pv + Pf	Pv + Pf	Pf	Pf	Pv + Pf	Pv + Pf	Pf	Pv + Pf	Pv + Pf	Pv + Pf
	2	Pf	Pf	Pf	Pf	Pf	Pf	Pf	Pf	Pf	Pv + Pf	Pv + Pf	Pv + Pf
	0.7	Pv + Pf	Pf	Pf	Pf	Pf	Pf	Pf	Pf	Pf	Pv + Pf	Pf	Pv + Pf
PCR positivity		18/24 (75%)^a,b^			15/24 (63%)^a^			17/24 (71%)^a^			23/24 (96%)^b^		
Target positivity		33/48 (69%)^a′^						40/48 (83%)^a′^					

Artificial mixtures containing *P. vivax* (Pv) and *P. falciparum* (Pf) in different proportions were prepared from well-characterized field samples, as described in Methods. The results are expressed as positive to *P. vivax* and *P. falciparum* (*Pv *+ *Pf*)*, P. vivax* (*Pv*) *or P. falciparum* (*Pf*). For each PCR assay, dilution points were assayed in triplicate (#1 to #3)

Different letters (a, b) indicate differences between proportions (*p *< 0.05, by Fisher’s exact test)

No difference was observed between proportions of targets positivity (a′)

### Field evaluation of ribosomal and non-ribosomal PCR targets in clinical and subclinical malaria infections

**Fig. 2:**
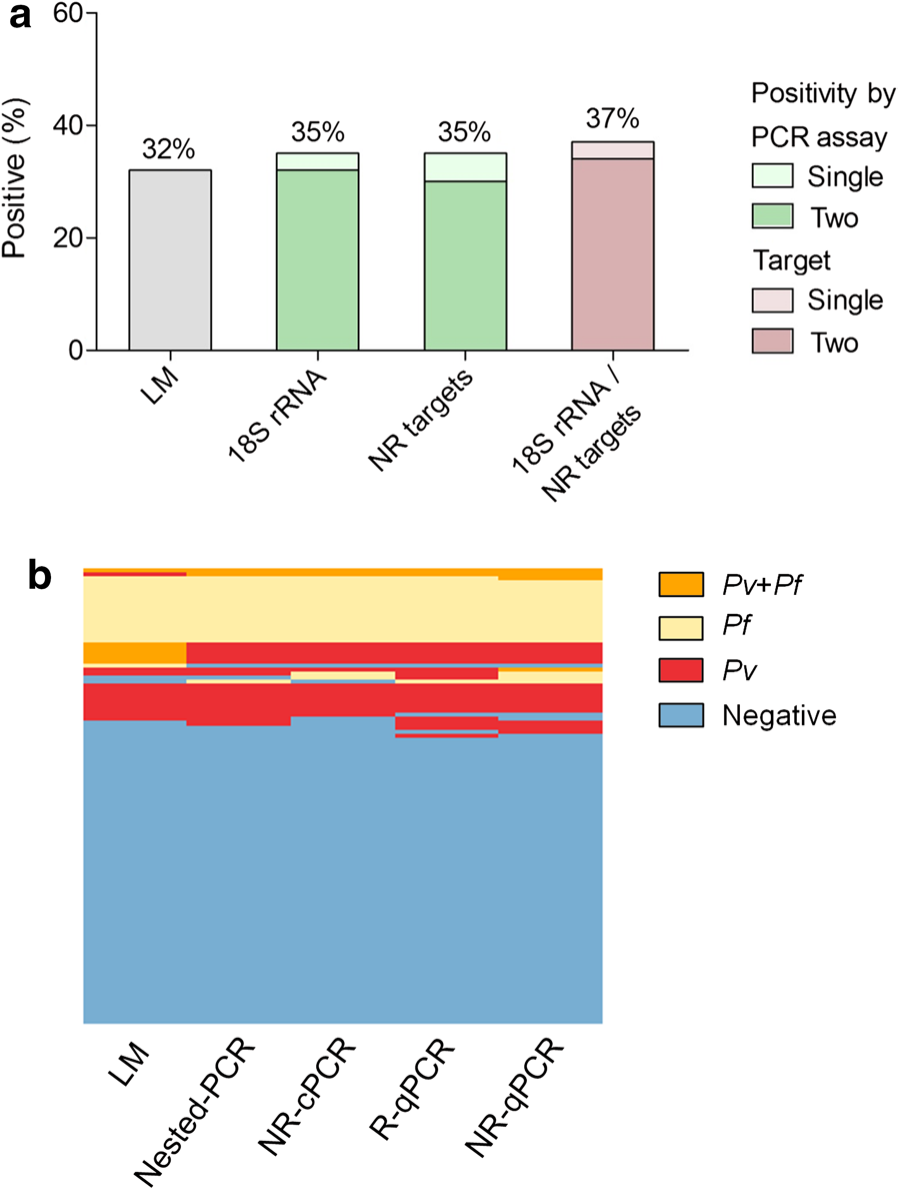
Malaria prevalence among clinical malaria suspects (n = 110) as detected by light microscopy (LM) or PCR-based protocols targeting ribosomal (*18S rRNA*) and non-ribosomal (NR) sequences of *P. vivax*/*P. falciparum.* The results were expressed as (**a**) frequency of positives according to the amplified parasite target region (*18S rRNA* and/or NR targets) or PCR assay (Nested-PCR vs. R-qPCR or NR-cPCR vs. NR-qPCR); p > 0.05 for all comparisons realized (Chi square test); (**b**) Heat map representation of species-specific positivity as detected by each PCR assay: blue—negative; red—*P. vivax*; yellow—*P. falciparum*; and orange—mixed *P. vivax/P. falciparum* infection. Each column represents an assay and subjects were represented in rows

Initially, the potential use of both ribosomal and non-ribosomal PCR targets for application in malaria field studies involved samples from 110 symptomatic individuals whose light microscopy (LM) confirmed 35 (32%) malaria infections (Fig. 2). As compared with LM, *18S rRNA* gene PCR-based assays (nested-PCR and/or R-qPCR) identified a similar proportion of malaria infections (35%, n = 39) (Fig. 2a); of interest, the majority of positive samples were amplified by both *18S rRNA* protocols. Basically, the same proportion of positives was obtained with the non-ribosomal protocols (NR-cPCR and/or NR-qPCR). In addition, the use of different PCR targets (ribosomal and non-ribosomal) did not increase malaria positivity (Fig. 2a, the right-side bar chart, in lilac; *p *= 1.0 for ribosomal vs. both PCR-targets and *p *= 0.89 for non-ribosomal vs. both targets). To confirm that an association of ribosomal and non-ribosomal targets did not increase sensitivity to detect submicroscopic malaria infections, the values of sensitivity and specificity were determined for molecular PCR-assays. In accordance, the performance of each PCR assay was similar with almost no detection of false positives or negatives (Table 5). *Plasmodium* species-specific identification (Fig. 2b) showed the same pattern of positivity, with no significant difference obtained between methods (LM vs. PCR based-protocols) or target amplified (ribosomal vs. non-ribosomal). In this symptomatic malaria patients, *P. vivax* and *P. falciparum* were found in similar proportions. Despite of that, a couple of mixed-infections (5 out of 6) identified by LM (Fig. 2b, first panel) could not be confirmed by any PCR-based assay.

**Table 5:** Assessment of sensitivity and specificity for molecular PCR-assays in clinical (n = 110) and subclinical (n = 324) malaria suspects

Study population/molecular protocol	True positive	False positive^a^	True negative	False negative	Sensitivity (CI 95%)	Specificity (CI 95%)
Clinical malaria suspects						
Nested-PCR	36	0	70	4	90% (0.76–0.97)	100% (0.95–1.0)
R-qPCR	37	1	70	2	95% (0.83–0.99)	99% (0.92–1.0)
NR-cPCR	34	0	70	6	85% (0.70–0.94)	100% (0.95–1.0)
NR-qPCR	36	1	70	3	92% (0.79–0.98)	99% (0.92–1.0)
Subclinical malaria suspects						
Nested-PCR	16	1	232	75	18% (0.10–0.27)	100% (0.98–1.0)
R-qPCR	67	2	232	23	74% (0.64–0.83)	99% (0.97–1.0)
NR-cPCR	42	0	232	50	46% (0.35–0.56)	100% (0.98–1.0)
NR-qPCR	72	17	232	3	96% (0.89–0.99)	93% (0.89–0.96)

Sensitivity and specificity was determined as previously described (23). The reference standard (true positive) for each protocol was calculated by combining the detections by any PCR, excluding the protocol under evaluation

^a^False positive in a sense that no other molecular protocol detected these infections

**Fig. 3:**
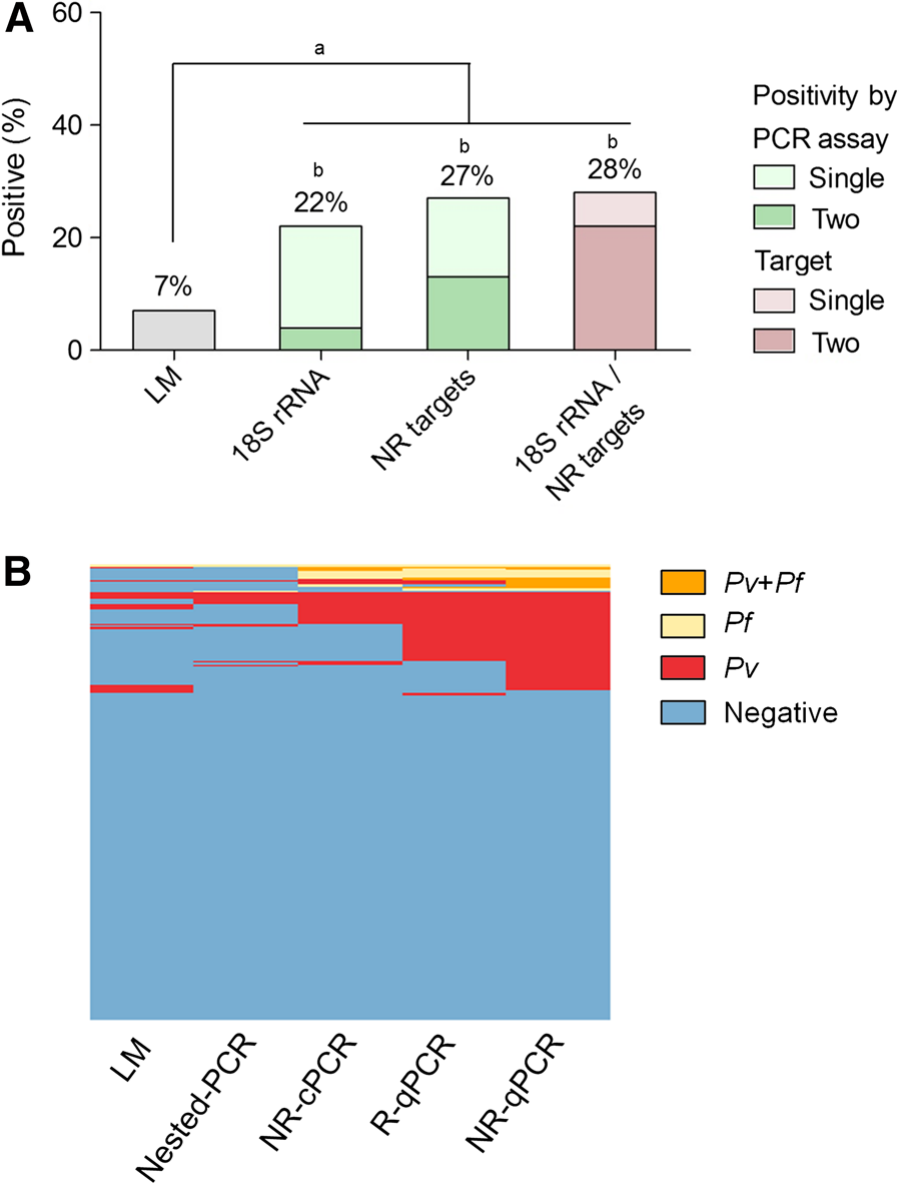
Malaria prevalence among subclinical malaria suspects (n = 324) as detected by Light Microscopy (LM) or PCR-based protocols targeting ribosomal (*18S rRNA*) and non-ribosomal (NR) sequences of *P. vivax* and *P. falciparum*. The results were expressed as (**A**) frequency of positives according to the amplified parasite target region (18SrRNA and/or NR targets) or PCR assay (nested-PCR vs. R-qPCR or NR-cPCR vs. NR-qPCR); Different letters (a, b) indicate differences between proportions (*p *< 0.05, Fisher’s exact approach for post hoc analysis of a Chi squared test); (**B**) Heat map representation of species-specific positivity as detected by each PCR protocols: blue—negative; red—*P. vivax*; yellow—*P. falciparum*; and orange—mixed *P. vivax*/*P. falciparum* infection. Each column represents an assay and subjects were represented in rows

**Fig. 4:**
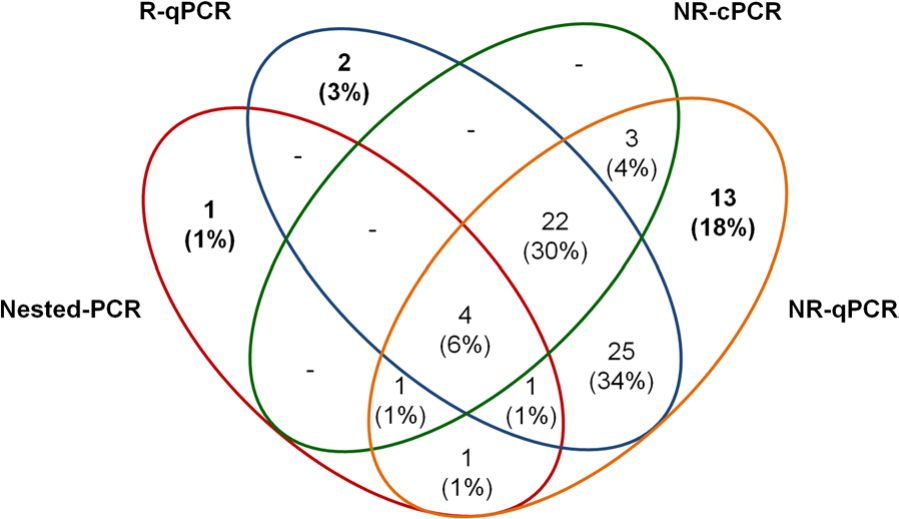
Venn diagram of submicroscopic malaria infections (n = 73) identified by each PCR-based assay targeting ribosomal (Nested-PCR and R-qPCR) and non-ribosomal (NR-cPCR and NR-qPCR). The results are shown as number of positive (%)

Screening for subclinical malaria infections among individuals from cross-sectional surveys identified a positivity of 7% (21 out of 324) by LM (Fig. 3A). In this population, molecular diagnosis of malaria increased three to four times the detection of subclinical malaria carries. However, the amplification of either *18S rRNA* gene (nested-PCR/R-qPCR) or non-ribosomal targets (NR-cPCR/NR-qPCR) detected a similar proportion of subclinical infections, being 22% (n = 72) and 27% (n = 89), respectively. As the predicted sensitivity of individual PCR assays varied significantly in this group of low-parasite densities, especially with high false negative rate for nested-PCR assay (Table 5), a significant amount of submicroscopic infections was identified only by the real-time PCR assays, i.e., R-qPCR for *18S rRNA* and NR-qPCR for Pvr47/Pfr364 sequences. In addition, the majority of submicroscopic infections were identified by both ribosomal and non-ribosomal targets (Fig. 3A, the right-side bar chart, in lilac; *p *= 0.09 ribosomal vs. both PCR-targets, and *p *= 0.86 for non-ribosomal vs. both targets). Overall, while PCR protocols identified 92 (28%) subclinical infections, only 21 (7%) were identified by LM (*p *< 0.0001, Fig. 3), indicating that 71 out of 92 (77%) infections were missed by the routine LM. Species-specific identification demonstrated a high proportion of *P. vivax* as compared with *P. falciparum* and mixed infections (Fig. 3B), and confirmed differences in the sensitivity between protocols. Although real-time PCR assays (R-qPCR and NR-qPCR) presented a better performance than conventional PCR assays (NRc-PCR and Nested-PCR), the results confirmed the gains achieved by using NR-qPCR to detect subclinical malaria infection (Fig. 3B). According, considering only the submicroscopic infections identified by any PCR-based assay (73 out of 92), Venn diagram demonstrated a high proportion of infections (18%, 13 out of 73) identified only by the NR-qPCR (Fig. 4). In terms of amplified target region, 96% (n = 70) of submicroscopic infections were detected by non-ribosomal sequences, while 78% (n = 57) were detected by ribosomal target (*p *= 0.0024, Fisher’s exact test).

## Discussion

Although major advances have been reached for the molecular detection of malaria parasites [25, 26, 36, 37], most sensitive PCR-based assays require high-volume of venous blood and complex sample processing [8, 23, 38, 39], being not feasible in the context of malaria routine surveillance. The current study involved investigate the hypothesis that the amplification of both ribosomal and non-ribosomal multi-copy PCR targets could increase the chances of detecting low parasite density and mixed *P. falciparum* and *P. vivax* infection. For that, a non-ribosomal (NR) qPCR targeting the multi-copy Pvr47/Pfr364 sequences was developed and this new protocol was compared with the original non-ribosomal gel-stained PCR-based protocol [24] as well as with two species-specific PCR assays based on the *18S rRNA* gene amplification.

The end-point titration assays of field samples revealed that the NR-qPCR protocol was able to accurately detect both *P. vivax* and *P. falciparum* infections—in single and artificial mixed infections—producing reproducible results at the lowest parasite densities (1–3 parasites/µL). Although there was considerable variation between PCR protocols assayed, the non-ribosomal protocols (NR-cPCR and NR-qPCR) were more accurate than ribosomal (nested-PCR and R-qPCR) to detect mixed-species infections. Of interest, only NR-qPCR assay developed here were able to detect *P. falciparum* when this species was present in a proportion of 240-fold lower than *P. vivax*. As the sensitivity of any PCR protocol depends largely on the molecular target used [40], the high copy number of Pfr364 (around 20 copies of “subfamily 1” targeted by specific primers) probably facilitated the detection of low levels of *P. falciparum* in co-infections as compared to *18S rRNA* (around 5–8 copies). Although different multi-copy targets have been described as sensitive for molecular diagnosis of malaria [23, 25, 36], those studies did not investigate the reliability of these targets in mixed-malaria infections, which precludes any potential comparison with results described here. In addition, most of the studies have been carried-out in endemic areas, such as Papua New Guinea, that currently does not represent an unstable and low-transmission endemic area [23]. More work needs to be done in this field of investigation. An apparent inability of *18S rRNA* qPCR to detect low *P. falciparum* densities in situation where *P. vivax* was present in much higher densities was observed. The use of a single pair of primers to detect both species may have been a determinant factor in causing failure of R-qPCR to identify mixed infections. A similar phenomenon of primer competition was described in the original protocol [17], straightening that species-specific primers should be used in field studies in which malaria co-infection is expected to be relevant.

In clinical malaria suspects, the overall prevalence for *P. vivax* and *P. falciparum* detected by amplification of non-ribosomal Pvr47/Pfr364 targets was not significantly different than that of either conventional microscopy or *18S rRNA* gene amplification. The predicted sensitivity and specificity of each PCR protocol assayed here were also similar, and it was independent of the parasite target. Although the clinical sample size limited the statistical power to detect small differences between protocols, these results were not completely unexpected as symptomatic patients usually present high parasite densities in the peripheral blood; consequently, it may facilitate the confirmation of malaria infection by less sensitive protocols such as microscopy and rapid diagnostic tests (RDTs) [41]. These findings reinforce that submicroscopic malaria infections are not prevalent among symptomatic patients, and LM and RDTs are adequate tools for case management [10, 42]. Nevertheless, the limited sensitivity of microscopy in correct identification of mixed-species malaria should be considered in areas where more than one *Plasmodium* species is circulating [43, 44], a result that was confirmed here.

While low frequencies of submicroscopic infections were observed in the group of clinical malaria cases (3–5%), screening for malaria in cross-sectional surveys demonstrated a large proportion (> 70%) of malaria cases in the study area that was not detected by conventional microscopy. The majority of subclinical infections were caused by *P. vivax,* the commonest malaria parasite in the Amazon basin, and frequently associated with low-density infections [27, 28, 45–47]. These findings are in accordance with recent reports showing high proportions of submicroscopic *P. vivax* infections across different endemic settings, particularly areas with relatively low transmission intensity [10–12, 26]. Although the reason for this high rate of asymptomatic *P. vivax* infections is unknown, it is probably associated with the unique biology of *P. vivax* that includes a fast acquisition of clinical immunity as compared with *P. falciparum* [47]. It is particularly relevant because in different epidemiological settings there are perspectives on treating asymptomatic infections for malaria elimination [48]. In the study area, the results demonstrated that, in general, multiple molecular targets (i.e., ribosomal plus non-ribosomal) did not increase sensitivity to detect subclinical malaria infections. Despite of that, the NR-qPCR developed here was the most sensitive protocol to detect submicroscopic asymptomatic malaria infections, which resulted in a significantly higher prevalence of submicroscopic infections (70 out of 73, 96%) when compared to that detected by ribosomal PCR assays (57 out of 73, 78%). While more sensitive amplification of *18S rRNA* gene has been described [39, 49], the likelihood of amplify *18S rRNA* gene was dependent on (i) large blood volume (2 mL); (ii) careful removal of plasma and buffy coat as prerequisite to avoid interference during PCR processing; (iii) concentration of purified DNA dehydrated in a centrifugal vacuum concentrator; additionally, these “high-volume” *18S rRNA* PCR strategy did not allow the detection by species (only *Plasmodium* spp.) [39].

The apparent ability of Pvr47/Pfr364 NR-qPCR to increase sensitivity to investigate the true prevalence of malaria infection is relevant as an unexpectedly large reservoir of infections may hinder control and elimination of malaria in the Americas [3, 50]. These findings are critical as both subclinical and submicroscopic malaria carriers remain untreated in the Brazilian Amazon region and therefore might remain infective over long periods of time [12]. As parasite densities cannot be assumed as a static parameter and thus may fluctuate over time falling below the detection threshold of the assay [51], future studies should approach longitudinal PCR-malaria surveys. Although the NR-qPCR developed here may constitute powerful additive tools to identify endemic sites where relevant control measures have to be settled and monitored [52], the costs of PCR-based assays limited such type of study. In general, nucleic acid amplification tests (NAATs) require expensive equipment available, well-equipped laboratories, qualified personnel, and large quantities of disposable supplies that need to be frozen or refrigerated, which is sometimes difficult in the country [41, 53]. Currently, WHO recommends that the use of NAATs be considered only for epidemiological research or surveys mapping submicroscopic infections in low transmission areas [54]. Innovative and cost-effective strategies that identify the real burden of malaria infections (those detected by qPCR) are required to reach malaria elimination goals, but remain a challenge [48].

Assuming that the NR-qPCR developed here seems to be the most sensitive method—as it was positive in a number of samples not detected by other PCR protocols—the results suggested that NR-qPCR has a lower detection threshold. Despite of that, it is important to clarify the technical limitations that apply for the definition of “reference standard” for PCR-detection of submicroscopic malaria infections. In general, the estimative of test accuracy are based on the assumptions that the reference standard is 100% sensitive and that specific disagreements between the reference standard and the diagnostic test being evaluated (index test) result from incorrect classification by the index test [55]. However, this statement cannot be applied for the detection of low-density malaria infections because there is no “gold-standard”. While the conventional microscopy diagnostic present high number of false negatives at low parasite density [41], there is no consensus about a PCR assay able to detect all malaria infections [25]. Due to these inherent limitations, the “reference standard” for each molecular diagnostic method was defined as a combination of positive detections by any PCR assay, excluding the method under evaluation, as described before [23]. Consequently, “false positive” in this type of analysis is considered in the sense that no other PCR method found these infections. In this scenario, the findings unlikely represent a tendency to false positive by NR-qPCR as it was established by (i) end-point titration of well-characterized field samples, including mono and artificial mixed-infections; (ii) reproducibility of replicates at low levels of parasitaemia; (iii) no amplification with gDNA samples from malaria-free volunteers; (iv) no cross-reactivity with other *Plasmodium* species. Furthermore, considering the rules for quality assessment of diagnostic accuracy studies (QUADAS-2) [56], the risk of bias of the present study was reduced as methodological design involved: (i) structured sample size calculations and random selection of malaria-exposed individuals, with explicit exclusion criteria defined in methods; (ii) in the estimative of sensitivity and/or specificity, the diagnostic test being evaluated was clearly interpreted before the reference standard was known; (iii) the execution of the PCR-based assays and the definition of reference standard were described in sufficient detail to permit replication of the test. Consequently, valuable malaria information can be retrieved from the current study.

Finally, relatively low frequencies of mixed-malaria infections were detected here, which precluded a more detailed evaluation of the potential of Pvr47/Pfr364 to detect mixed-malaria infections in the field. In the Amazon area, besides *P. vivax* being the predominant malaria parasite [27, 29], the progress achieved in malaria control has decreased the number of *P. falciparum* cases in recent years [57]. Notwithstanding this study limitation, it is highly relevant the results from the end-point titration experiments showing the ability of Pvr47/Pfr364 to consistently detect *P. vivax/P. falciparum* co-infection, as the accurate detection of malaria mixed-infections seems to be critical for control and management of malaria [43, 44]. In fact, disease burden due to mixed species infections remains largely unknown, and this limitation have the potential to influence decisions on testing vaccines and new antimalarial drugs [58]. As malaria has been re-emerging in areas where it was previously controlled, dealing with mixed malaria infection cannot be bypassed, as recent evidence suggest that the frequency of these infections may be much higher than previously expected [44], including in the Amazon region [59]. Due to the risk of *P. falciparum* reemergence from Amazonian neighboring countries with high transmission rates, a cross-border malaria study to evaluate the relevance of NR-qPCR in mixed-malaria infections are on progress.

## Conclusion

Although the simultaneous use of ribosomal and non-ribosomal PCR-targets did not impact the molecular diagnosis of malaria, the amplification of Pfr364 and Pvr47 multi-copy targets by the NR-qPCR seems to be a valuable tool in detection of subclinical and mixed *P. vivax/P. falciparum* infections, even though one of the species was present in a ratio of hundred-times lower than the other species.

## Additional files

**Additional file 1.** Analytical evaluation of NR-qPCR assay.

**Additional file 2.** Amplification curves of NR-qPCR performed on field samples infected with *P. vivax* (n = 3; red), *P. falciparum* (n = 3; blue), *P. malariae* (n = 3; green), and *P. brasilianum* DNA (n = 1; orange). The amplification plots are shown for **(A)** Pvr47 and **(B)** Pfr364 assays.

**Additional file 3.** Five-fold dilution of *P. vivax*-Pvr47 and *P. falciparum*-Pfr364 plasmids amplified by NR-qPCR.

**Additional file 4.** Conditions of the Nested-PCR, R-qPCR, and NR-cPCR assays. The primers/probes used for *P. vivax* and *P. falciparum* targets were the original described. The products of nested-PCR and NR-cPCR were visualized by 2% agarose gel stained with ethidium bromide.

**Additional file 5.** Limit of detection (LOD) for **(A)** Pvr47 and **(B)** Pfr364 targets amplified by NR-qPCR. Probit regression analysis was used on logarithmic scale using nine points of NR-qPCR standard curves (20,000 to 0.05 copies/μL). The calculated regression curves (blue lines) indicate the probability (y-axis) of obtaining positive results at any template concentration, and dashed brown lines shows 95% confidence intervals. Dashed black lines correspond to the lower DNA concentration in which 95% of positive samples were detected (0.66 copies/µL of Pvr47 and 3.27 copies/µL of Pfr364).

## Abbreviations

18S rRNA: small subunit 18S of the ribosomal RNA gene
NR: non-ribosomal
PCR: polymerase chain reaction
NR-qPCR: non-ribosomal real-time PCR
NR-cPCR: non-ribosomal conventional PCR
R-qPCR: ribosomal real-time PCR
IQR: interquartile range
gDNA: genomic DNA
LM: light microscopy
LOD: limit of detection

## References

[1] WHO. World Malaria Report 2018. Geneva: World Health Organization. 2018. https://apps.who.int/iris/bitstream/handle/10665/275867/9789241565653-eng.pdf?ua=1. Accessed 9 Apr 2019.

[2] Wassmer SC, Grau GER (2017). Severe malaria: what’s new on the pathogenesis front?. Int J Parasitol.

[3] Recht J, Siqueira AM, Monteiro WM, Herrera SM, Herrera S, Lacerda MVG (2017). Malaria in Brazil, Colombia, Peru and Venezuela: current challenges in malaria control and elimination. Malar J..

[4] Rabinovich RN, Drakeley C, Djimde AA, Hall BF, Hay SI, Hemingway J (2017). malERA: an updated research agenda for malaria elimination and eradication. PLoS Med..

[5] Imwong M, Stepniewska K, Tripura R, Peto TJ, Lwin KM, Vihokhern B (2016). Numerical distributions of parasite densities during asymptomatic malaria. J Infect Dis.

[6] Tadesse FG, Van Den Hoogen L, Lanke K, Schildkraut J, Tetteh K, Aseffa A (2017). The shape of the iceberg: quantification of submicroscopic *Plasmodium falciparum* and *Plasmodium vivax* parasitaemia and gametocytaemia in five low endemic settings in Ethiopia. Malar J..

[7] Lamptey H, Ofori MF, Kusi KA, Adu B, Yeboa EO, Baafour EK (2018). The prevalence of submicroscopic *Plasmodium falciparum* gametocyte carriage and multiplicity of infection in children, pregnant women and adults in a low malaria transmission area in Southern Ghana. Malar J..

[8] Bejon P, Andrews L, Hunt-cooke A, Sanderson F, Gilbert SC, Hill AVS (2006). Thick blood film examination for *Plasmodium falciparum* malaria has reduced sensitivity and underestimates parasite density. Malar J..

[9] Haanshuus CG, Chandy S, Manoharan A, Vivek R (2016). A high malaria prevalence identified by PCR among patients with acute undifferentiated fever in India. PLoS ONE.

[10] Cheng Q, Cunningham J, Gatton ML (2015). Systematic review of sub-microscopic *P. vivax* infections: prevalence and determining factors. PLoS Negl Trop Dis..

[11] Moreira CM, Shehada MA, Price RN, Drakeley CJ (2015). A systematic review of sub-microscopic *Plasmodium vivax* infection. Malar J..

[12] Almeida ACG, Kuehn A, Castro AJM, Vitor-Silva S, Figueiredo EFG, Brasil LW (2018). High proportions of asymptomatic and submicroscopic *Plasmodium vivax* infections in a peri-urban area of low transmission in the Brazilian Amazon. Parasit Vectors..

[13] Alves FP, Gil LHS, Marrelli MT, Ribolla PEM, Camargo EP, Pereira Da Silva LH (2005). Asymptomatic carriers of *Plasmodium* spp. as infection source for malaria vector mosquitoes in the Brazilian Amazon. J Med Entomol..

[14] Schneider P, Bousema JT, Gouagna LC, Otieno S, van de Vegte-Bolmer M, Omar SASR (2007). Submicroscopic *Plasmodium falciparum* gametocyte densities frequently result in mosquito infection. Am J Trop Med Hyg.

[15] Chen I, Clarke SE, Gosling R, Hamainza B, Killeen G, Magill A (2016). “Asymptomatic” malaria: a chronic and debilitating infection that should be treated. PLoS Med..

[16] Snounou G, Viriyakosol S, Zhu PZ, Jarra W, Pinheiro L, Rosario VE (1993). High sensitivity of detection of human malaria parasites by the use of nested polymerase chain reaction. Mol Biochem Parasitol..

[17] Rougemont M, Van Saanen M, Sahli R, Hinrikson HP, Bille JJK (2004). Detection of four *Plasmodium* species in blood from humans by 18S rRNA gene subunit-based and species-specific real-time PCR assays. J Clin Microbiol.

[18] Mangold KA, Manson RU, Koay ES, Stephens L, Regner M, Thomson RB, Peterson LRKK (2005). Real-time PCR for detection and identification of *Plasmodium* spp. J Clin Microbiol.

[19] Murphy SC, Prentice JL, Williamson K, Wallis CK, Fang FC, Fried M (2012). Real-time quantitative reverse transcription PCR for monitoring of blood-stage *Plasmodium falciparum* infections in malaria human challenge trials. Am J Trop Med Hyg.

[20] Kamau E, Alemayehu S, Feghali KC, Saunders D, Ockenhouse CF (2013). Multiplex qPCR for detection and absolute quantification of malaria. PLoS ONE.

[21] Mercereau-Puijalon O, Barale J, Bischoff E (2002). Three multigene families in *Plasmodium* parasites: facts and questions. Int J Parasitol.

[22] Costa DC, Madureira AP, Amaral LC, Sanchez BAM, Gomes LT, Fernandes Fontes CJ (2014). Submicroscopic malaria parasite carriage: how reproducible are polymerase chain reaction-based methods?. Mem Inst Oswaldo Cruz.

[23] Hofmann NE, Gruenberg M, Nate E, Ura A, Rodriguez-Rodriguez D, Salib M (2018). Assessment of ultra-sensitive malaria diagnosis versus standard molecular diagnostics for malaria elimination: an in-depth molecular community cross-sectional study. Lancet Infect Dis..

[24] Demas A, Oberstaller J, DeBarry J, Lucchi NW, Srinivasamoorthy G, Sumari D (2011). Applied genomics: data mining reveals species-specific malaria diagnostic targets more sensitive than 18S rRNA. J Clin Microbiol.

[25] Hofmann N, Mwingira F, Shekalaghe S, Robinson LJ, Mueller I, Felger I (2015). Ultra-sensitive detection of *Plasmodium falciparum* by amplification of multi-copy subtelomeric targets. PLoS Med..

[26] Gruenberg M, Moniz CA, Hofmann NE, Wampfler R, Koepfli C, Mueller I (2018). *Plasmodium vivax* molecular diagnostics in community surveys: pitfalls and solutions. Malar J..

[27] Kano FS, Sanchez BAM, Sousa TN, Tang ML, Saliba J, Oliveira FM (2012). *Plasmodium vivax* Duffy binding protein: baseline antibody responses and parasite polymorphisms in a well-consolidated settlement of the Amazon Region. Trop Med Int Health..

[28] Souza-silva A, Brito CFA, Adams JH, Kano FS, Carvalho LH (2014). Duffy antigen receptor for chemokine (DARC) polymorphisms and its involvement in acquisition of inhibitory anti-duffy binding protein II (DBPII) immunity. PLoS ONE.

[29] Kano FS, Souza-Silva FA, Torres LM, Lima BA, Sousa TN, Alves JR, Rocha RS, Fontes CJ, Sanchez BA, Adams JH, Brito CF, Pires DE, Ascher DB, Sell AMCL (2016). The presence, persistence and functional properties of *Plasmodium vivax* duffy binding protein II antibodies are influenced by HLA class II allelic variants. PLoS Negl Trop Dis..

[30] Ladeia-Andrade S, Ferreira MU, de Carvalho ME, Curado I, Coura JR (2009). Age-dependent acquisition of protective immunity to malaria in riverine populations of the Amazon Basin of Brazil. Am J Trop Med Hyg.

[31] Pires CV, Alves JRS, Lima BAS, Paula RB, Sousa N, Soares IS (2018). Blood-stage *Plasmodium vivax* antibody dynamics in a low transmission setting: a nine year follow-up study in the Amazon region. PLoS ONE.

[32] Ministério da Saúde. Manual de Diagnóstico Laboratorial da Malária. Brasília: Secretaria de Vigilância em Saúde. 2009. http://bvsms.saude.gov.br/bvs/publicacoes/manual_diagnostico_laboratorial_malaria_2ed.pdf. Accessed 9 Apr 2019.

[33] Ministério da Saúde. Guia prático de tratamento da malária no Brasil. Brasília: Secretaria de Vigilância em Saúde. 2010. http://bvsms.saude.gov.br/bvs/publicacoes/guia_pratico_malaria.pdf. Accessed 9 Apr 2019.

[34] Olsson ML, Hansson C, Avent ND, Åkesson IE, Green CA, Daniels GL (1998). A clinically applicable method for determining the three major alleles at the Duffy (FY) blood group locus using polymerase chain reaction with allele-specific primers. Transfusion..

[35] Trager W, Jensen JB (1976). Human malaria parasites in continuous culture. Science.

[36] Lloyd YM, Esemu LF, Antallan J, Thomas B, Yunga ST, Obase B (2018). PCR-based detection of *Plasmodium falciparum* in saliva using mitochondrial cox3 and varATS primers. Trop Med Health..

[37] Meerstein-Kessel L, Andolina C, Carrio E, Mahamar A, Sawa P (2018). A multiplex assay for the sensitive detection and quantification of male and female *Plasmodium falciparum* gametocytes. Malar J..

[38] Andrews L, Andersen RF, Webster D, Dunachie S, Walther RM, Bejon P (2005). Quantitative real-time polymerase chain reaction for malaria diagnosis and its use in malaria vaccine clinical trials. Am J Trop Med Hyg.

[39] Imwong M, Hanchana S, Malleret B, Rénia L, Day NPJ, Dondorp A (2014). High-throughput ultrasensitive molecular techniques for quantifying low-density malaria parasitemias. J Clin Microbiol.

[40] Lucchi NW, Oberstaller J, Kissinger JCUV (2013). Malaria diagnostics and surveillance in the post-genomic era. Public Health Genomics..

[41] Zimmerman PA, Howes RE (2015). Malaria diagnosis for malaria elimination. Curr Opin Infect Dis..

[42] Bousema T, Okell L, Felger I, Drakeley C (2014). Asymptomatic malaria infections: detectability, transmissibility and public health relevance. Nat Rev Microbiol.

[43] Ehtesham R, Fazaeli A, Raeisi A, Keshavarz H, Heidari A (2015). Detection of mixed-species infections of *Plasmodium falciparum* and *Plasmodium vivax* by nested PCR and rapid diagnostic tests in southeastern Iran. Am J Trop Med Hyg.

[44] Singh US, Siwal N, Pande V, Das A (2017). Can mixed parasite infections thwart targeted malaria elimination program in India?. Biomed Res Int.

[45] Oliveira-ferreira J, Lacerda MVG, Brasil P, Ladislau JLB, Tauil PL, Daniel-ribeiro CT (2010). Malaria in Brazil: an overview. Malar J..

[46] Sampaio VS, Siqueira AM, Costa G, Paula M, Mourão G, Monteiro WM (2015). Malaria in the State of Amazonas: a typical Brazilian tropical disease influenced by waves of economic development. Rev Soc Bras Med Trop.

[47] Adams JH, Mueller I (2017). The biology of *Plasmodium vivax*. Cold Spring Harb Perspect Med..

[48] Jaiteh F, Masunaga Y, Okebe J, Alessandro UD, Balen J, Bradley J (2019). Community perspectives on treating asymptomatic infections for malaria elimination in The Gambia. Malar J..

[49] Imwong M, Nguyen TN, Tripura R, Peto TJ, Lee SJ, Lwin KM (2015). The epidemiology of subclinical malaria infections in South-East Asia: findings from cross-sectional surveys in Thailand–Myanmar border areas, Cambodia, and Vietnam. Malar J..

[50] Ferreira MU, Castro MC (2016). Challenges for malaria elimination in Brazil. Malar J..

[51] Nguyen T, Seidlein LV, Nguyen T, Truong P, Hung SD, Pham H (2018). The persistence and oscillations of submicroscopic *Plasmodium falciparum* and *Plasmodium vivax* infections over time in Vietnam: an open cohort study. Lancet Infect Dis..

[52] Niang M, Diop F, Niang O, Sadio BD, Sow A, Faye O (2017). Unexpected high circulation of *Plasmodium vivax* in asymptomatic children from Kédougou, southeastern Senegal. Malar J..

[53] Berzosa P, De Lucio A, Barja MR, Herrador Z, González V, García L (2018). Comparison of three diagnostic methods (microscopy, RDT, and PCR) for the detection of malaria parasites in representative samples from Equatorial Guinea. Malar J..

[54] WHO. Nucleic acid amplification-based diagnostics. Geneva: World Health Organization. 2018. https://www.who.int/malaria/areas/diagnosis/nucleic-acid-amplification-tests/en/. Accessed 9 Apr 2019.

[55] Biesheuvel C, Irwig L, Bossuyt P (2007). Observed differences in diagnostic test accuracy between patient subgroups: is it real or due to reference standard misclassification?. Clin Chem..

[56] Whiting PF, Rutjes AWS, Westwood ME, Mallett S, Deeks JJ, Reitsma JB (2011). QUADAS-2: a revised tool for the quality assessment of diagnostic accuracy studies. Ann Intern Med.

[57] Siqueira AM, Mesones-lapouble O, Marchesini P, Sampaio VDS, Brasil P, Tauil PL (2016). *Plasmodium vivax* landscape in Brazil: scenario and challenges. Am J Trop Med Hyg.

[58] Zimmerman PA, Mehlotra RK, Kasehagen LJKJ (2004). Why do we need to know more about mixed *Plasmodium* species infections in humans?. Trends Parasitol..

[59] Camargo M, León SCS, Del Río-ospina L, Páez AC, González Z, González E (2018). Micro-epidemiology of mixed-species malaria infections in a rural population living in the Colombian Amazon region. Sci Rep..

