# Supplementary material for: Ribosomal and non-ribosomal PCR targets for the detection of low-density and mixed malaria infections
Source: Malar J. 2019 Apr 30;18:154. doi: 10.1186/s12936-019-2781-3 (PMC6492410; doi:10.1186/s12936-019-2781-3)
Supplement: Supplementary file 1 — Additional file 1. Analytical evaluation of NR-qPCR assay. [file 12936_2019_2781_MOESM1_ESM.pdf]

Additional File 1. Analytical evaluation of NR-qPCR assay

Specificity of NR-qPCR was confirmed both *in silico* (BLAST search) and experimentally by using genomic DNA from 30 healthy volunteers (malaria-free) whose samples were not amplified for either Pvr47 or Pfr364 targets (data not shown). For assessment of cross-reactivity with other *Plasmodium* species, a panel of *Plasmodium* reference samples (as described in material and methods) was submitted to the NR-qPCR assay. Non-specific amplification was observed by using either pooled samples from *P. vivax* (n=3), *P. falciparum* (n=3), *P. malariae* (n=3), or an sample from *P. brasilianum* (Fig. S1). Recombinant plasmids containing Pvr47 or Pfr364 sequences were constructed to determine the limit of detection (LOD) of each specie-specific DNA target. For that, a conventional PCR was performed in two separate reactions using the same primers for amplification of Pvr47 and Pfr364 by NR-qPCR, both performed in a 20 µL mixture containing 1.0 µM of each primer, 0.6 µL of Taq DNA polymerase (Invitrogen), 125 µM of dNTP (Invitrogen), 2 µL of 10X Buffer (Invitrogen), 0.75 mM of $MgCl_2$ (Invitrogen), and 2 µL of DNA. PCR conditions consisted of an initial denaturation at 94°C for 3 min followed by 35 cycles of 94°C for 20 sec, 58°C for 30 sec, and 72°C for 30 sec. A final extension of 72°C for 2 min was included and all amplifications were performed on a Veriti Thermal Cycler (Applied Biosystems). The amplicons were inserted into a pGEM-T Easy vector (Promega) according to manufacturer's instructions. Isolation of recombinant plasmids was performed with QIAprep Miniprep (Qiagen), and the presence of the inserts was confirmed by 2% agarose gel after digestion with EcoRI (Promega). The purified plasmids were linearized with ApaI (Promega) and quantified in a Qubit 2.0 Fluorometer (Life Technologies). The plasmid numbers were estimated as follows: *number of plasmid copies* = ((6.02 × 10^{23} *copies/mol*) × *amount of DNA (g)*) /

(length of DNA (bp) × 660 (g/mol/bp)) (26). Serial dilutions of plasmids were prepared at five-fold dilutions ranged from 20,000 to 0.05 copies/µL (Table S1). By using probit regression analysis, the NR-qPCR assay presented a 95% probability of detecting levels as low as 0.66 copies/µL for *P. vivax* (Fig. S2A) and 3.27 copies/µL for *P. falciparum* (Fig. S2B).
