# Supplementary material for: Ribosomal and non-ribosomal PCR targets for the detection of low-density and mixed malaria infections
Source: Malar J. 2019 Apr 30;18:154. doi: 10.1186/s12936-019-2781-3 (PMC6492410; doi:10.1186/s12936-019-2781-3)
Supplement: Supplementary file 2 — Additional file 2. Amplification curves of NR-qPCR performed on field samples infected with P. vivax (n = 3; red), P. falciparum (n = 3; blue), P. malariae (n = 3; green), and P. brasilianum DNA (n = 1; orange). The amplification plots are shown for (A) Pvr47 and (B) Pfr364 assays. [file 12936_2019_2781_MOESM2_ESM.pdf]

**Additional File 2.** Amplification curves of NR-qPCR performed on field samples infected with *P. vivax* (n=3; red), *P. falciparum* (n=3; blue), *P. malariae* (n=3; green), and *P. brasilianum* DNA (n=1; orange). The amplification plots are shown for **(A)** Pvr47 and **(B)** Pfr364 assays.

**A**

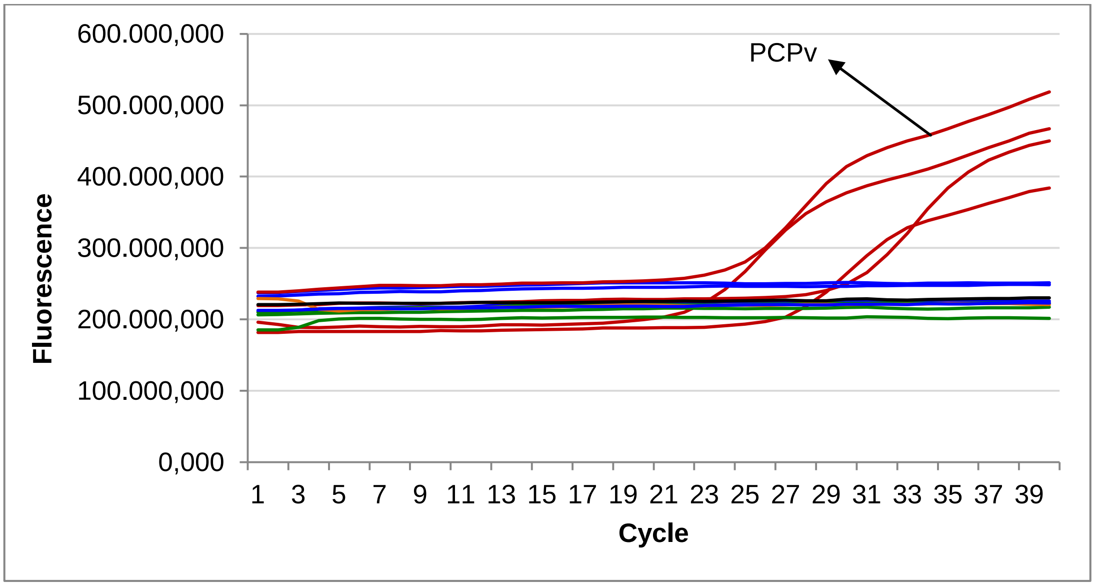


**B**

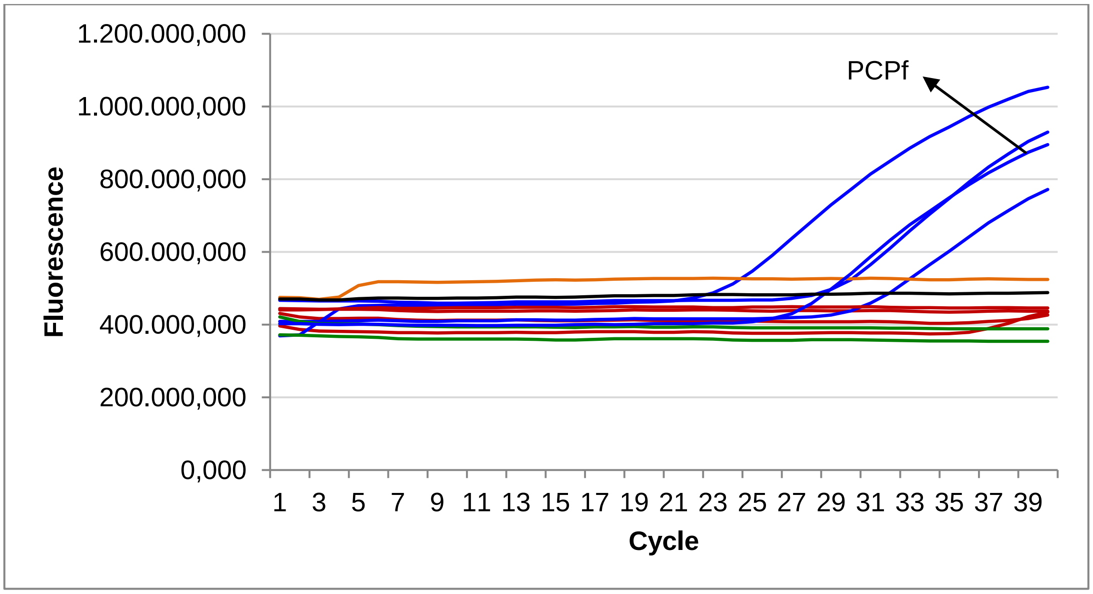


PCPv = positive control of *P. vivax*; PCPf = positive control of *P. falciparum*.
