# Supplementary material for: Ribosomal and non-ribosomal PCR targets for the detection of low-density and mixed malaria infections
Source: Malar J. 2019 Apr 30;18:154. doi: 10.1186/s12936-019-2781-3 (PMC6492410; doi:10.1186/s12936-019-2781-3)
Supplement: Supplementary file 3 — Additional file 3. Five-fold dilution of P. vivax-Pvr47 and P. falciparum-Pfr364 plasmids amplified by NR-qPCR. [file 12936_2019_2781_MOESM3_ESM.pdf]

Additional File 3. Five-fold dilution of *P. vivax*-Pvr47 and *P. falciparum*-Pfr364 plasmids amplified by NR-qPCR

**Plasmid**	***P. vivax*-Pvr47**			
**Copies/μL DNA***	**Positive/n° replicates**		**Mean C_t (Ct SD)**	
	**Exp 1**	**Exp 2**	**Exp 1**	**Exp 2**
20,000	3/3	NP	24.3 (0.04)	NP
4,000	3/3	NP	26.7 (0.07)	NP
800	3/3	3/3	29.0 (0.03)	29.3 (0.12)
160	3/3	3/3	31.2 (0.22)	30.9 (0.24)
32	5/5	5/5	33.0 (1.40)	33.5 (0.26)
6.4	5/5	5/5	34.9 (0.40)	34.4 (0.60)
1.28	5/5	5/5	36.8 (1.20)	33.5 (1.28)
0.25	3/5	5/5	38.0 (0.60)	32.8 (0.60)
0.05	2/5	5/5	35.5 (1.05)	34.4 (1.13)
**Plasmid**	***P. falciparum*-Pfr364**			
**Copies/μL DNA***	**Positive/n° replicates**		**Mean C_t (Ct SD)**	
	**Exp 1**	**Exp 2**	**Exp 1**	**Exp 2**
20,000	3/3	NP	23.1 (0.05)	NP
4,000	3/3	NP	25.4 (0.10)	NP
800	3/3	3/3	27.7 (0.10)	28.3 (0.12)
160	3/3	3/3	29.9 (0.06)	30.7 (0.42)
32	5/5	5/5	32.4 (0.40)	32.7 (0.40)
6.4	5/5	5/5	34.5 (0.60)	34.7 (1.06)
1.28	4/5	3/5	36.5 (1.00)	36.6 (0.55)
0.25	4/5	2/5	37.1 (0.20)	36.2 (0.45)
0.05	0/5	0/5	NA	NA

*2μL of template DNA were added per PCR reaction. Two independent experiments were performed (Exp 1 and Exp 2), and three to five replicates per dilution point. NP=not performed. NA=not applicable.
