# Supplementary material for: Ribosomal and non-ribosomal PCR targets for the detection of low-density and mixed malaria infections
Source: Malar J. 2019 Apr 30;18:154. doi: 10.1186/s12936-019-2781-3 (PMC6492410; doi:10.1186/s12936-019-2781-3)
Supplement: Supplementary file 4 — Additional file 4. Conditions of the Nested-PCR, R-qPCR, and NR-cPCR assays. The primers/probes used for P. vivax and P. falciparum targets were the original described. The products of nested-PCR and NR-cPCR were visualized by 2% agarose gel stained with ethidium bromide. [file 12936_2019_2781_MOESM4_ESM.pdf]

Additional File 4. Conditions of the Nested-PCR, R-qPCR, and NR-cPCR assays. The primers/probes used for *P. vivax* and *P. falciparum* targets were the original described. The products of nested-PCR and NR-cPCR were visualized by 2% agarose gel stained with ethidium bromide.

**Target**	18S rRNA	18S rRNA	Pvr47/Pfr364
**Protocol***	Nested-PCR	R-qPCR	NR-cPCR
**PCR reaction conditions**			
Final volume	20 µL	12.5 µL	10 µL
DNA template	0.8 µL	2 µL	1 µL
Primers (fwd and rev)	250 nM	200 nM	500 nM
Probe	-	80 nM	-
2X PCR Master Mix	10 µL	-	-
Taqman Universal PCR Master Mix 2X (Applied Biosystems)	-	6.25 µL	-
Deoxyribonucleotide triphosphates (dNTP)	-	-	125 µM
Taq DNA polymerase (Invitrogen)	-	-	0.3 µL
10X Buffer	-	-	1 µL
$MgCl_2$	-	-	0.75 mM
**PCR cycling conditions**			
Initial step	95°C – 5 min<br>58°C – 2 min<br>72°C – 2 min	50ºC – 2 min<br>95ºC – 10 min	95°C – 2 min
Denaturation, annealing and elongation	94ºC – 1 min<br>58ºC – 2 min<br>72ºC – 2 min	95ºC – 15 sec<br>60ºC – 1 min	95°C – 30 sec<br>58°C – 30 sec<br>72°C – 45 sec
Final extension	58ºC – 2 min<br>72ºC – 5 min	-	72°C – 5 min
Number of cycles	24**	45	35
**Platform**	PTC-100TM v.7.0 (MJ Research Inc, USA) and Veriti Thermal Cycler (Applied Biosystems)	7500 Real-Time PCR System (Applied Biosystems)	Veriti Thermal Cycler (Applied Biosystems)

*Nested-PCR (14; 20), R-qPCR (15), and NR-qPCR (22).

******The cycling parameters for the second round were the same as the first reaction, but with 29 cycles of amplification.
