# Supplementary material for: Ribosomal and non-ribosomal PCR targets for the detection of low-density and mixed malaria infections
Source: Malar J. 2019 Apr 30;18:154. doi: 10.1186/s12936-019-2781-3 (PMC6492410; doi:10.1186/s12936-019-2781-3)

Additional File 5. Limit of detection (LOD) for **(A)** Pvr47 and **(B)** Pfr364 targets amplified by NR-qPCR. Probit regression analysis was used on logarithmic scale using nine points of NR-qPCR standard curves (20,000 to 0.05 copies/µL). The calculated regression curves (blue lines) indicate the probability (y-axis) of obtaining positive results at any template concentration, and dashed brown lines shows 95% confidence intervals. Dashed black lines correspond to the lower DNA concentration in which 95% of positive samples were detected (0.66 copies/µL of Pvr47 and 3.27 copies/µL of Pfr364).

A

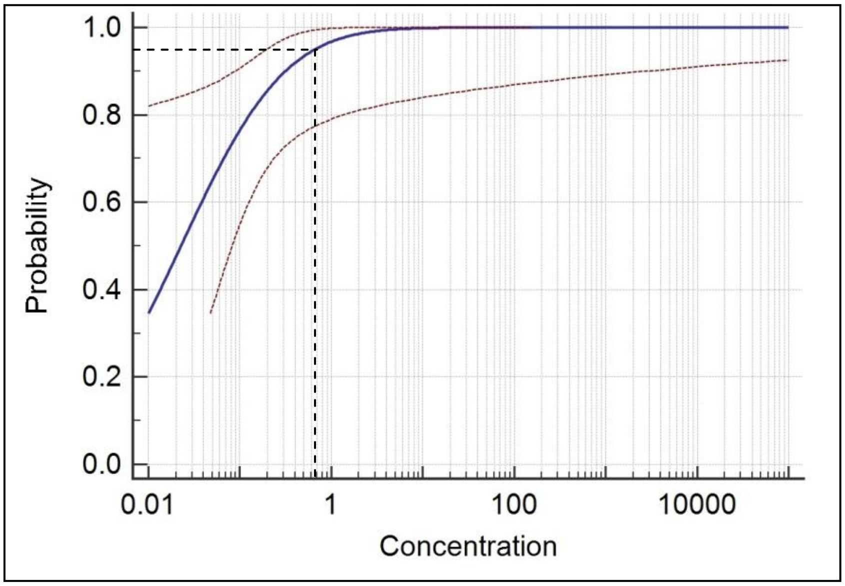

B

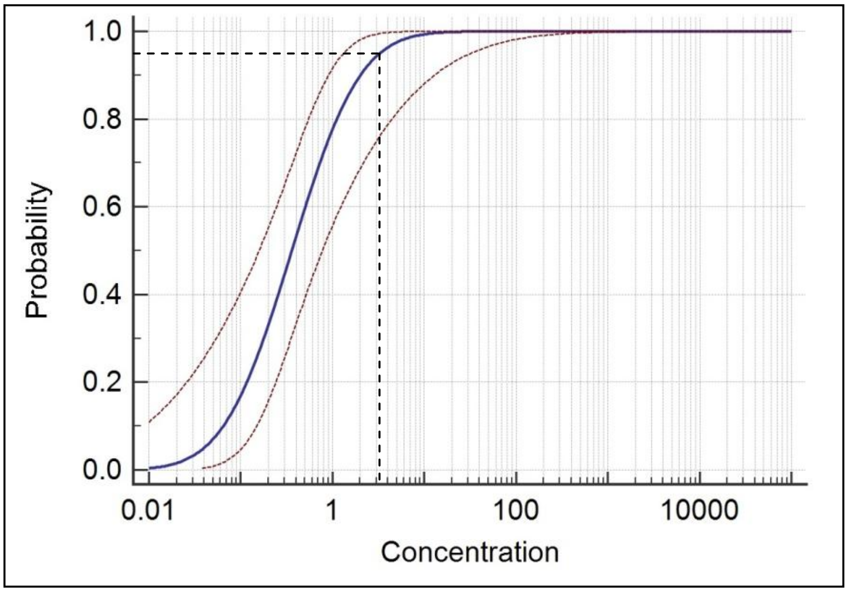

Supplement: Supplementary file 5 — Additional file 5. Limit of detection (LOD) for (A) Pvr47 and (B) Pfr364 targets amplified by NR-qPCR. Probit regression analysis was used on logarithmic scale using nine points of NR-qPCR standard curves (20,000 to 0.05 copies/μL). The calculated regression curves (blue lines) indicate the probability (y-axis) of obtaining positive results at any template concentration, and dashed brown lines shows 95% confidence intervals. Dashed black lines correspond to the lower DNA concentration in which 95% of positive samples were detected (0.66 copies/µL of Pvr47 and 3.27 copies/µL of Pfr364). [file 12936_2019_2781_MOESM5_ESM.pdf]
